# The Role of Interventional Radiology and Management of an Angiography Suite in the Treatment of COVID-19 Patients: Single-Center, 2-Year Experience

**DOI:** 10.3390/medicina59020188

**Published:** 2023-01-17

**Authors:** Jongjoon Shim, Jae Myeong Lee

**Affiliations:** Department of Radiology, Soonchunhyang University Bucheon Hospital, Bucheon 14584, Republic of Korea

**Keywords:** interventional radiology, angiography room, COVID-19, disinfection

## Abstract

*Background and Objectives*: Hospital angiography suites with negative-pressure ventilation facilities are challenging to equip. During the COVID-19 pandemic, we aimed to introduce interventional radiology procedures performed on COVID-19 patients and understand management of the angiography suite without a negative-pressure ventilation facility before and after the procedures to prevent the spread of infection. *Materials and Methods*: Between December 2020 and November 2022, 52 COVID-19 patients underwent interventional radiology procedures in an angiography suite, where no negative-pressure ventilation facility was installed. During the procedure, all staff members wore full personal protection equipment, and after the procedure for the COVID-19-positive patient was completed, the angiography suite was disinfected and entry to the angiography suite was prohibited for 1–3 h. In this angiography suite, procedures for COVID-19 patients and non-COVID-19 patients were performed. *Results*: A total of 61 interventional radiology procedures were performed in 52 patients with COVID-19. Of 52 patients, 21 underwent procedures under intubation and mechanical ventilation. All procedures were performed according to the guidelines set by the Infection Control Committee of our hospital. No major or minor complications were associated with the procedures. There were no cases of infection among staff members or other non-COVID-19 patients related to procedures on COVID-19 patients. *Conclusions*: Interventional radiology can play an important role in solving the complications of COVID-19 and the problems caused by patients’ underlying diseases. In addition, if accurate guidelines are followed, both COVID-19 and non-COVID-19 patients can undergo procedures in an angiography room without negative-pressure ventilation while preventing infection.

## 1. Introduction

Coronavirus disease 2019 (COVID-19) was first reported in Wuhan, China, in December 2019 [1]. COVID-19 is a severe and fatal respiratory infection caused by severe acute respiratory syndrome coronavirus 2 (SARS-CoV-2), suspected to originate from an endemic bat virus [2,3,4]. On 11 March 2020, the World Health Organization (WHO) declared the COVID-19 pandemic [5]. SARS-CoV-2 has been confirmed to be transmitted from person to person via several means, including droplets, aerosols, and vectors [6]. SARS-CoV-2 enters human cells through angiotensin-converting enzyme 2 (ACE2) receptors found in the lungs, heart, kidneys, intestines, and vascular endothelium [7].

While COVID-19 is mostly mild, about 20% of COVID-19 patients may develop a severe illness [8]. Patients infected with SARS-CoV-2 initially show flu- or cold-like symptoms, such as dry cough, sore throat, fever, fatigue, muscle aches, headache, abdominal pain, and diarrhea, but eventually develop partial opacities in the lungs, respiratory depression, and death [9,10,11,12,13].

The estimated mortality rate of COVID-19 is 2% [14]. However, the prognosis is poor when patients with underlying diseases are infected with SARS-CoV-2 [15]. In addition, patients with severe COVID-19 may develop complications such as respiratory distress, renal failure, cardiac injury, and venous and arterial thromboembolism [16,17]. Interventional radiology plays an important role in resolving the problems caused by underlying diseases or complications of COVID-19 patients.

The COVID-19 pandemic has also affected the field of interventional radiology [18]. In the context of the COVID-19 pandemic, the interventional radiology department has made many preparations and achieved significant achievements [19,20,21,22,23].

When treating COVID-19 patients, not only the success of the procedure, but also the prevention of the spread of infection, is very important. Therefore, when performing interventional procedures for COVID-19 patients, a facility equipped with negative-pressure ventilation is required [19,20,21,22,23]. However, with the rapid spread of infection, it is difficult to equip all hospital angiography suites with negative-pressure ventilation facilities.

There have been some reports of interventional procedures performed on patients with COVID-19 in the early stages of the pandemic [20,21,22,23]. However, these studies consisted only of a short-term follow-up early during the pandemic. To the best of our knowledge, there have been no studies with more than one year of experience in performing interventional procedures in patients with COVID-19. In addition, there have been no reports on the preparation process of the angiography suite before the procedure for such patients or the disinfection process after the procedure.

Although our hospital’s angiography suite was not equipped with a negative-pressure ventilation facility, both COVID-19 and non-COVID-19 patients underwent procedures in this angiography suite during the pandemic. In this study, we would like to introduce the interventional procedures performed on COVID-19 patients during the two-year pandemic and the pre-procedure preparation as well as post-procedure disinfection process of an angiography suite without a negative-pressure ventilation facility.

## 2. Materials and Methods

### 2.1. Patients

This single-center, retrospective study was approved by the institutional review board of our hospital. The requirement for informed consent was waived owing to the retrospective nature of the study.

We retrospectively reviewed the medical records of 119 patients with COVID-19 who underwent interventional procedures between December 2020 and November 2022. The purpose of this study was to introduce interventional procedures performed on COVID-19 patients and analyze infection control in an angiography suite without a negative pressure ventilation facility. Therefore, 67 patients who underwent procedures at the bedside or operating room equipped with a negative-pressure ventilation facility were excluded.

Of the 119 patients, 52 underwent interventional procedures in an angiography suite without a negative-pressure ventilation facility, and 52 patients (male: female = 33:19, age: 69 ± 14.2) were included in this study.

### 2.2. Preprocedural Evaluation

The COVID-19 status was confirmed prior to the interventional radiology procedure using a reverse transcriptase-polymerase chain reaction (RT-PCR) assay to detect the presence of SARS-CoV-2 RNA in nasopharyngeal swab specimens. In addition, after reviewing medical records, we investigated the underlying diseases of COVID-19 patients.

Additionally, chest computed tomography (CT) was performed in all patients to confirm pneumonia caused by SARS-CoV-2.

### 2.3. Preparation of Angiography Suite

Interventional radiology procedures for COVID-19 patients were performed after all procedures for non-COVID-19 patients were completed, unless it was an emergency. Access to the angiography suite by nonessential personnel was strictly controlled prior to performing the procedures in patients with COVID-19. Objects not required for the procedure that could have been contaminated with patient droplets in the angiography suite were removed whenever possible. Instruments or objects that could not be removed were covered to prevent droplet contamination.

### 2.4. Personal Infection Prevention Measures and Patient Transportation

The operators and all personnel involved in the procedure wore full personal protective equipment (PPE). PPE is placed in the changing room within the angiography suite, and this changing room is not a particularly secluded area. Full PPE consists of surgical gown, gloves, surgical cap, face shield, or goggles and mask (KF94). Due to the specificity of the angiography suite where radiation occurs, all personnel wore a lead apron under the surgical gown.

**Figure 1:**
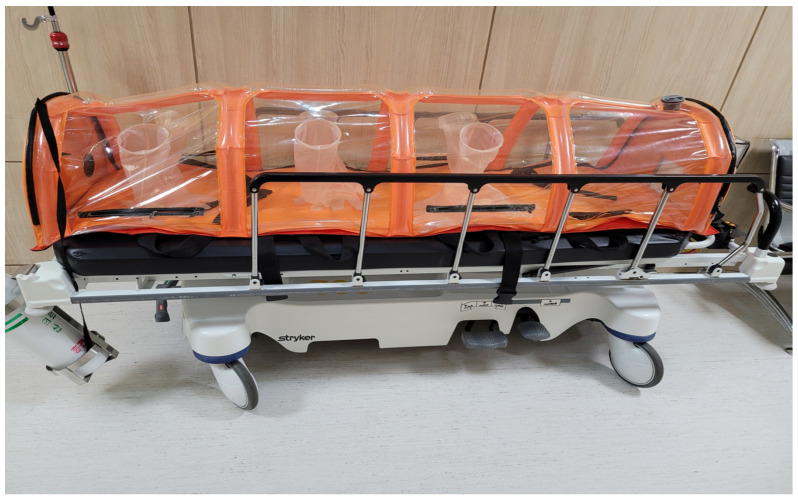
COVID-19 patients are transported using this tent when moving from the ward to the angiography suite.

After the preparation of the angiography suite and staff members, the patient was transferred from the ward to angiography suite. When transporting the patients, people were prohibited from entering the transport route, and the patients were transported to the angiography suite using an isolation tent (Figure 1).

### 2.5. Interventional Radiology Procedure Process

When a COVID-19 patient was transported to the angiography suite, staff members wearing full PPE opened the isolation tent and moved the patient to the operating table. After wiping the inside and outside of the tent that carried the patient with a tissue containing benzalkonium chloride (SafeCide; Cosell Care, Hanam, Republic of Korea), the tent was moved to a space outside the angiography suite.

The interventional radiology procedures were performed by two experienced interventional radiologists. Operators and assistants directly participating in the procedure performed the procedure wearing lead goggles, surgical cap that covered the ears, KF94 mask, face shield, sterilized surgical gown, and sterilized surgical gloves.

For venous catheter insertion, such as central venous infusion catheter, hemodialysis catheter, and peripherally inserted central venous catheter (PICC), veins such as the internal jugular vein, subclavian vein, and upper arm basilic or brachial vein were punctured under ultrasonography (US) guidance, and catheterization was performed using the Seldinger technique.

The insertion of a drainage catheter for drainage of pleural effusion or peritoneal fluid collection was also performed using the Seldinger technique after puncturing the fluid site under US guidance.

In the case of percutaneous cholecystostomy (PTGBD) or percutaneous transhepatic biliary drainage, the gallbladder or intrahepatic bile duct was punctured with a 21G needle under US guidance, and a drainage catheter was inserted into the gallbladder or bile duct using the Seldinger technique.

Arterial angiography or transcatheter arterial embolization was performed by puncturing the right or left common femoral artery under US guidance, followed by insertion of a 5-F introducer sheath (Terumo, Tokyo, Japan) and catheterization of a 5-F angiographic catheter (Cobra, Cook Medical, Bloomington, IN, USA or Rosch Hepatic, Cook Medical, Bloomington, IN, USA) and a microcatheter (Progreat, Terumo, Tokyo, Japan) into the target vessel.

### 2.6. Disinfection Process after the Procedure

After the procedure, the patient was moved to an isolation tent. After the patient completely left the angiography suite, the equipment in contact with the patient, such as a US probe and operating table, was disinfected with 1000 ppm sodium hypochlorite solution. After disinfection of equipment, PPE was removed, and PPE removal was performed in the procedure room.

The removal of PPE and disposal of waste should be performed meticulously. The order of PPE removal is as follows: 1. removal of gloves and gown, 2. hand hygiene, 3. wipe face shield using benzalkonium chloride tissue, 4. hand hygiene 5. remove the face shield, 6. hand hygiene, 7. remove the surgical cap, 8. hand hygiene, 9. put on a mask, and 10. hand hygiene.

Biological waste, including PPE, droplet protection covers, and procedure instruments, was double-wrapped and disposed of in biohazard bags. Contaminants that could not be discarded, such as lead goggles or lead aprons, were disinfected by wiping them with benzalkonium chloride.

During the process of removing PPE or disposing of contaminated waste, angiography suite staff members frequently sanitized their hands using a 70% ethanol gel. After disposal of the contaminated waste and disinfection of the angiography suite, entry into the angiography suite was prohibited for 3 h.

**Figure 2:**
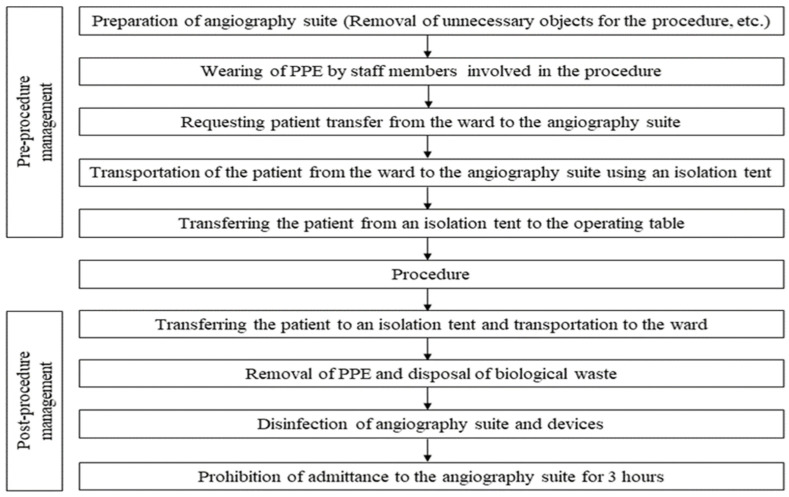
The process and sequence of interventional radiologic procedures in patients with COVID-19.

Figure 2 summarizes the sequence and process from the preparation of the angiography suite before the procedure to the management of the angiography suite after the procedure.

### 2.7. Follow-Up

Medical records of all 52 patients were reviewed to confirm the occurrence of complications related to interventional radiology procedures. Operators and staff members who participated in the procedures performed on patients with COVID-19 measured body temperature every day for one week after the procedure and checked for symptoms, such as coughing or sore throat. In addition, confirmation of transmission of infection to non-COVID-19 patients who underwent the procedure in the angiography suite was performed by reviewing patients’ medical records and investigating and reporting the occurrence of nosocomial infections in our hospital by the infection control team.

### 2.8. Definition

A recent study revealed that the mean latent period of COVID-19 is 5.5 days [24]. Therefore, staff member infections related to procedures in patients with COVID-19 were defined as cases in which symptoms such as fever, cough, and sore throat occurred within 7 days of participation in the procedure and SARS-CoV-2 RNA was detected by RT-PCR.

Procedure-related infection between patients was defined as cases in which symptom onset and RT-PCR results were positive within 7 days in non-COVID-19 patients who underwent the procedure in the angiography suite within 1 day after the COVID-19 patient underwent the procedure.

Procedure-related complications were classified according to the Society of Interventional Radiology (SIR) Adverse Event Classification System [25]: A, no therapy, no consequences; B, nominal therapy, no consequence, including overnight admission for observation only; C, requires therapy, minor hospitalization (<48 h); D, requires major therapy, unplanned increase in level of care, prolonged hospitalization (>48 h); E, permanent adverse sequelae; and F, death. We defined category A and B complications as minor. Complications in categories C, D, E, and F were defined as major complications.

## 3. Results

In all 52 patients, SARS-CoV-2 RNA was detected by RT-PCR performed at our hospital or external hospitals prior to the interventional radiology procedure. In addition, all patients underwent chest CT, and lung opacity suggestive of pneumonia was observed.

Patient demographics and clinical characteristics are summarized in Table 1. 46 patients had underlying disease. Twenty-five patients had two or more underlying diseases. The most common medical comorbidities were hypertension (*n* = 27), diabetes (*n* = 19), and chronic renal disease (*n* = 11). Twenty-one patients underwent procedures under intubation and mechanical ventilation. The median hospitalization period was 33 days (range: 4–167 days) and median follow-up period was 52 days (range: 5–357 days). During the follow-up period, 33 patients were discharged from hospital and 19 patients died.

A total of 61 interventional radiology procedures were performed on 52 patients. The types of interventional radiology procedures performed on patients are summarized in Table 2. All 61 procedures were performed under local anesthesia. There were no cases where the procedure was performed under general anesthesia.

The most frequently performed interventional radiology procedure was central venous catheter insertion (24.5%). Hemodialysis catheter insertion was performed in 13 cases (21.3%). In 5 of these 13 cases, hemodialysis catheter insertion was performed because acute renal failure occurred in patients without prior renal disease. PTGBD was performed on 12 patients (19.7%), and all cases were acalculous cholecystitis. Two patients had acute thromboembolism in the right iliac artery; therefore, transcatheter mechanical thrombectomy and intra-arterial stent insertion were performed. The occluded arteries were recanalized in both patients.

Most procedures for patients with COVID-19 were assigned and performed in the last order of the procedure schedule for the day. However, urgent cases, such as transcatheter arterial embolization, were assigned as an emergency and performed prior to the remaining procedures, even though there were still procedures to be performed in the angiography suite. Twelve procedures were performed in this way, and the angiography suite was prepared and disinfected before and after the procedure as mentioned above. The remaining procedures for non-COVID-19 patients were performed after the angiography suite was closed for 1 h according to the guidelines of the infection control committee of our hospital.

No major or minor complications were associated with interventional radiology procedures performed on patients with COVID-19. Two interventional radiologists, four nurses, and three radiologic technologists participated in the procedures for these patients. From the time the procedure for patients with COVID-19 was first started in our hospital’s angiography suite to the time of reporting this study, there were no cases of participating staff members being infected by patients during the procedure. In addition, there were no cases of SARS-CoV-2 infection in non-COVID-19 patients who underwent procedures in the angiography suite after the procedures for COVID-19 patients.

## 4. Discussion

At the beginning of the COVID-19 pandemic, the number of people infected with SARS-CoV-2 in the Republic of Korea was relatively low compared with that in other countries [26]. Therefore, in the early phase of the pandemic, the management and treatment of COVID-19 patients were carried out only at national or public hospitals. These national or public hospitals were quickly equipped with negative-pressure ventilation facilities through government support and served exclusively as dedicated hospitals for COVID-19 patients during the pandemic. Therefore, despite being a tertiary university hospital, our hospital transferred patients to dedicated COVID-19 hospitals when such patients reported in the early phase of the pandemic.

However, since August 2020, the number of patients with COVID-19 has increased, causing a shortage of beds in national and public hospitals [26]; therefore, some private tertiary hospitals have participated in treating these patients. Therefore, in November 2020, our hospital also created a dedicated ward for patients with COVID-19 having 10 beds equipped with a negative-pressure ventilation facility. Since July 2021, the number of these patients in Korea has increased more rapidly [26], and from November 2021, our hospital has increased the number of beds dedicated to patients with COVID-19 to 50.

In the early days, when we performed procedures on patients with COVID-19, there were very few procedures, most of which were relatively simple, such as central venous catheter insertion or pleural fluid drainage. Therefore, in the early days, procedures for patients with COVID-19 were performed under portable US or portable fluoroscopy guidance on the bedside or in a strictly isolated and controlled dedicated operating room equipped with a negative-pressure ventilation facility to prevent the spread of infection.

However, as the number of hospitalized patients with COVID-19 increased, the procedures performed on these patients diversified, resulting in limitations in performing procedures at the bedside or operating room. Therefore, in consultation with the infection control committee of our hospital, guidelines for conducting procedures in the angiography suite for patients with COVID-19 were created and implemented.

Patients with COVID-19 often have underlying diseases which can affect the prognosis of COVID-19 [15]. Therefore, in addition to pneumonia, complications caused by underlying diseases in these patients also require treatment, and interventional radiology plays an important role in this.

COVID-19 can affect many parts of the body besides the lungs, causing various complications [16,17]. Renal complications are also common in patients with COVID-19, with acute kidney injury occurring in 36.6% of hospitalized patients, among which 14.3% required renal replacement therapy [27]. In our study, five patients had acute renal failure and underwent hemodialysis catheter insertion. Large-bore catheter insertion, such as a hemodialysis catheter, can cause serious complications such as central vein rupture if not performed under accurate US and fluoroscopic guidance. Therefore, hemodialysis catheterization should be performed by an experienced interventional radiologist in the angiography suite.

COVID-19 can cause vascular thromboembolism [28]. We also performed transcatheter mechanical thrombectomy in two patients with COVID-19 who developed arterial thromboembolism.

We performed PTGBD in 12 cases and all cases were acute acalculous cholecystitis. Acute acalculous cholecystitis is characterized by an acute necroinflammatory disease of the gallbladder without gallstones [29]. Acalculous cholecystitis is also strictly related to other pathological conditions such as trauma, cardiopulmonary resuscitation, mechanical ventilation, sepsis, burn, prolonged total parenteral nutrition, and major surgery [30]. It accounts for only 10% of acute cholecystitis cases [31], but severely ill patients with COVID-19 undergoing mechanical ventilation may have a relatively high incidence. Surgical cholecystectomy is generally performed to treat acute acalculous cholecystitis [32]. However, surgery for patients with COVID-19 requires many staff members, and the process can be complicated. In addition, for some patients with poor general condition or a high risk of surgery under general anesthesia, surgery can be very risky or even impossible. PTGBD can be performed with only local anesthesia without general anesthesia, and the procedure time and process are very simple compared to surgical cholecystectomy.

Of the 52 patients with COVID-19 who underwent interventional radiology procedures, 19 died. These 19 patients died due to exacerbation of respiratory distress caused by pneumonia, although the complications were resolved with the procedures. The mortality rate in patients undergoing the procedure may be high. However, as our hospital is a tertiary referral hospital, it is necessary to consider that patients with severe COVID-19 are hospitalized and treated.

As noted above, interventional radiology procedures can play an important role in managing COVID-19 complications or problems that may arise from patients’ underlying diseases. However, the role of interventional radiology in treating patients with COVID-19 has been underestimated.

Some institutions have installed and operated negative-pressure ventilation facilities in angiograph suites [21], but many hospitals have not yet been able to do so. Our hospital also did not have a negative-pressure ventilation facility applied to the angiography suite. In addition, since there is only one angiography suite used by the interventional radiology department, it was not possible to operate it as a dedicated facility for patients with COVID-19.

The installation of a digital subtraction angiography machine is complex, space consuming, and very expensive. Therefore, it is very difficult to change the structure of the angiography suite or install it additionally within a short period during the infectious disease pandemic. Hospitals in developing countries, in particular, may face more difficulties. Therefore, it is necessary to understand how to deal with a pandemic of infectious diseases with existing facilities and equipment.

During an infectious disease pandemic, interventional radiologists and staff members participating in the procedure should be familiar with the procedure as well as the spread of pathogens and disinfectants [33]. We found no cases of infection of staff members, including operators, or of other patients who underwent procedures after patients with COVID-19 due to droplet blocking and appropriate disinfection procedures.

Our study had some limitations. The number of procedures performed on 52 patients over two years may seem small. Due to the retrospective study design, the study had to be conducted by reviewing medical records. In particular, the confirmation of the occurrence of infection in other non-COVID-19 patients was made only by medical record review and the report of the infection control team at our hospital; therefore, there was a limit to confirming the accurate spread of infection. In addition, since most procedures rarely induce aerosol, accurate analysis of prevention of aerosol diffusion is difficult.

As mentioned above, 67 procedures performed at the bedside or operating room equipped with a negative-pressure ventilation facility were excluded. Patients whose infection status was uncertain or who were released from isolation after treatment for infection were excluded from the study. The aforementioned process was also applied to these patients. Our study was performed only on patients who had clearly tested positive for COVID-19 and had very high infectivity. Thus, the number of procedures associated with COVID-19 patients was far greater than those mentioned in this study.

Previous studies [19,20,21,22,23] were conducted at the beginning of the pandemic and focused on managing of the angiography suite. During the two-year COVID-19 pandemic, our study was the first to investigate the spread of infection to staff members or other patients involved in interventional radiology procedures. We hope that our study will be helpful to hospitals in many countries that have not yet established clear management and procedural guidelines during the pandemic of infectious diseases.

## 5. Conclusions

Interventional radiology is not an unrelated field during a pandemic of infectious diseases. Interventional radiology plays an important role in solving problems caused by underlying diseases or complications caused by infectious diseases. If the correct guidelines are set and the procedure is performed, the procedure can be safely performed while preventing infection in an angiography suite that does not have a negative-pressure ventilation facility and is not exclusively for COVID-19 patients, as in our study. Therefore, it is necessary to establish and implement guidelines for managing an angiography suite in which interventional radiology procedures are performed during an infectious disease pandemic.

## Figures and Tables

**Table 1:** Patients’ demographics and clinical characteristics.

Age (Years)	Mean	68.1
	Range	27–94
Gender	Male	33 (63.5%)
	Female	19 (36.5%)
Number of underlying diseases	None	6 (11.5%)
	One	21 (40.4%)
	More than two	25 (48.1%)
Types of underlying diseases	HTN	27
	DM	19
	CKD	11
	Cancer	7
	LC	1
	Others	7
Respiratory state	Spontaneous respiration	31
	Mechanical ventilation	21
Follow-up period (days)	Median	52
	Range	5–357

HTN, hypertension; DM, diabetes mellitus; CKD, chronic kidney disease; LC, liver cirrhosis.

**Table 2:** Types of interventional radiology procedures.

	*n*, % (*n* = 61)
Central venous catheter insertion	15 (24.5%)
Hemodialysis catheter insertion	13 (21.3%)
Percutaneous cholecystostomy	12 (19.7%)
Percutaneous catheter drainage	7 (11.5%)
Transcatheter arterial embolization	7 (11.5%)
Percutaneous mechanical thrombectomy	2 (3.3%)
Percutaneous transhepatic biliary drainage	2 (3.3%)
Percutaneous nephrostomy	2 (3.3%)
Percutaneous transluminal angioplasty	1 (1.6%)

## Data Availability

The datasets generated during and/or analyzed during the current study are available from the corresponding author on reasonable request.
